# Full Spectrum Electrochromic WO_3_ Mechanism and Optical Modulation via Ex Situ Spectroscopic Ellipsometry: Effect of Li^+^ Surface Permeation

**DOI:** 10.3390/mi15121473

**Published:** 2024-12-05

**Authors:** Buyue Zhang, Jintao Wang, Shuhui Jiang, Meng Yuan, Xinyu Chen

**Affiliations:** 1 School of Physics, Changchun University of Science and Technology, Changchun 130012, China; zhangbuyue729@163.com; 2 School of Infromation Engineering, Yantai Institute of Technology, Yantai 261499, China; 3 School of Materials Science and Engineering, Jilin Architecture University, Changchun 130119, China; 4 Key Laboratory of Functional Materials Physics and Chemistry of the Ministry of Education, Jilin Normal University, Changchun 130012, China

**Keywords:** WO_3_, electrochromic, protocol of ex situ methods, ex situ spectroscopic, ellipsometry

## Abstract

Tungsten oxide (WO_3_) electrochromic devices are obtaining increasing interest due to their color change and thermal regulation. However, most previous work focuses on the absorption or transmission spectra of materials, rather than the optical parameters evolution in full spectrum in the electrochromic processes. Herein, we developed a systematic protocol of ex situ methods to clarify the evolutions of subtle structure changes, Raman vibration modes, and optical parameters of WO_3_ thin films in electrochromic processes as stimulated by dosage-dependent Li^+^ insertion. We obtained the below information by ex situ spectroscopic ellipsometry. (1) Layer-by-layer Li^+^ embedding mechanism demonstrated by individual film thickness analysis. (2) The details of its optical leap in the Brillouin zone in the full spectral. (3) The optical constants varied with the Li^+^ insertion in the ultraviolet, visible, and near-infrared bands, demonstrating the potential for applications in chip fabrication, deep-sea exploration, and optical measurements. (4) Simulated angular modulation laws of WO_3_ films for full spectra in different Li^+^ insertion states. This ex situ method to study the optical properties of electrochromic devices are important for monitoring phase transition kinetics, the analysis of optical leaps, and the study of ion diffusion mechanisms and the stoichiometry-dependent changes in optical constants over the full spectral. This work shows that electrochromic films in Li^+^ surface permeation can be applied in the field of zoom lenses, optical phase modulators, and other precision optical components. Our work provides a new solution for the development of zoom lenses and a new application scenario for the application of electrochromic devices.

## 1. Introduction

In recent years, adaptive lenses with zoomable capability have attracted attention for their wide range of applications in correction of ophthalmic lens [1,2], zoom lens [3,4], aberration compensation [5,6], biomedical imaging [7,8,9], optical tweezers [10,11], beam steering [12,13], fiber coupling [14,15], and so on. These zoom lenses are usually realized based on three techniques [16]. The first is based on an optical system constructed with multiple sets of optical elements, where the focal length is changed by a nonlinear movement of a spherical or cylindrical lens along the optical axis [17]. The second is the liquid crystal lens [18]; the main working principle is to adjust the refractive index of liquid crystal to realize the change in focal length. The third is the liquid lens [19], the main way is through the external force changes the shape of the lens to realize the focal length change. Replacing the moving optics with a stationary adaptive lens can simplify the system, reduce the size and power consumption, and eliminate vibration caused by conventional mechanic movement [20].

In recent years, tungsten oxide (WO_3_) electrochromic devices have attracted much attention in the direction of color change and thermal regulation; it has broad application prospects in smart windows [21], low-power displays [22], antiglare rearview mirrors [23], smart textiles [24], energy storage systems [25], heat-shielding systems [26], sensing [27], catalysis and military camouflage, etc. These properties are based on the properties defined by changes in the absorption or transmission spectra of WO_3_ films. The ion insertion process changes the crystal structure of the WO_3_ films and causes a change in the optical constants [28]. This indicated the possibility of electrochromic technology applied in the field of adaptive lenses. In this paper, we have used WO_3_, a classical electrochromic system, as a template to capture the refractive index changes in the full spectral of the film during lithium-ion (Li^+^) insertion using a series of ex situ measurement techniques. The refractive angle modulation law of the Li^+^ insertion dosage-dependent at full spectrum is analyzed.

In this work, we prepared γ-WO_3_ by sol–gel spin-coating, and the film gradually shifted from transparent to dark blue with the layer-by-layer embedding of Li^+^. The samples were tested by ex situ X-ray diffraction (XRD), ex situ Raman spectroscopy, and ex situ spectroscopic ellipsometry during the coloration process. The results of ex situ XRD and ex situ Raman spectroscopy demonstrate that the crystal structure of the samples gradually transforms the γ → β → α-phase during the coloration process. We obtained the optical constants in the full spectral of the samples in different lithium-ion insertion dosage states by ex situ spectroscopic ellipsometry. The Li^+^ insertion into the γ-WO_3_ samples is a layer-by-layer insertion mechanism, as can be seen from the results of the thickness variation in each film layer. And we fitted a quantitative relationship between the Li^+^ insertion dosage (x) and the Li_x_WO_3_ layer thickness. The detail of all optical leap points in the Brillouin zone corresponding to the range of 1–5 eV is also found. The change in extinction coefficient (k) with Li^+^ insertion shows that its band gap decreases from 3.22 eV to 3.14 eV. The results of its refractive index (*n*) in the ultraviolet, visible, and near-infrared bands show obvious peak motion and peak shape changes, which demonstrates the difference in its *n* adjusting ability for specific wavelengths of light and shows the prospect of its application in the fields of chip processing, undersea exploration, and optical inspection. Our work provides a new solution for the development of precision optical components such as zoom lenses, as well as a new application scenario for the application of electrochromic devices.

## 2. Results

### 2.1. Ex Situ XRD Monitored Structure Evolution in Electrochromic Process

**Figure 1:**
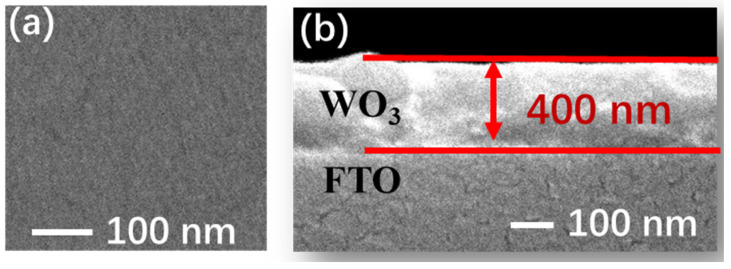
(**a**) Planar and (**b**) cross-sectional photographs of field emission scanning electron microscopy of WO_3_ thin film. The as-fabricated WO_3_ film is composed of uniformly distributed small particles that are difficult to discern with a diameter smaller than 10 nm. The as-coated WO_3_ layer shows an average thickness of 380 nm uniformly and compactly on the top surface of the ITO layer.

The as-fabricated WO_3_ film is composed of uniformly distributed small particles that are difficult to discern with a diameter smaller than 10 nm, according to the SEM in Figure 1a. The as-coated WO_3_ layer shows an average thickness of 380 nm uniformly and compactly on the top surface of the ITO layer in Figure 1b. The as-prepared WO_3_ film crystallized into a typical monoclinic crystalline system (γ-phase) as indicated in JCPDS card No. of 18–0376.

With the insertion of lithium, the peaks of WO_3_ at 23.1° and 24.3° are shifted toward a small angle due to the monoclinic structure of WO_3_ toward an increasing symmetry [29]. It has been widely reported that for WO_3_ with temperature, in bulk form, phase transformation occurs in the following sequence: monoclinic II (ε-WO_3_, T < −43 °C) → triclinic (δ-WO_3_, −43 °C < T < 17 °C) → monoclinic I (γ-WO_3_, 17 °C < T < 330 °C) →orthorhombic (β-WO_3_, 330 °C < T < 740 °C) → tetragonal (α-WO_3_, T > 740 °C) [30] For the lithium insertion process in this work, the structure undergoes a similar change: γ → β → α; we listed the details of the specific peaks in Table 1.

**Figure 2:**
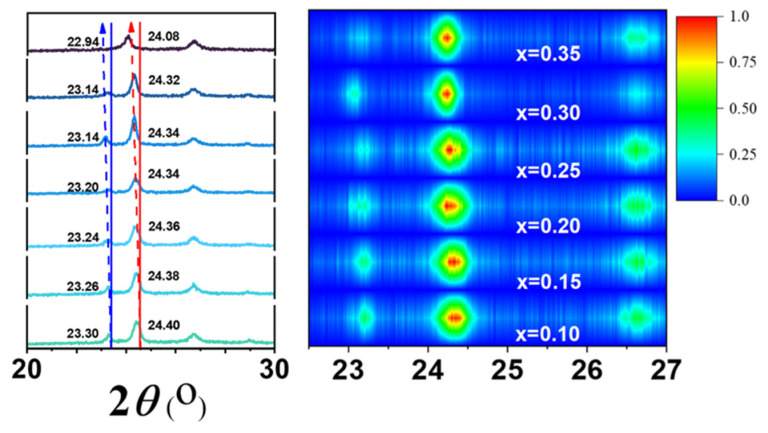
The XRD results of the Li_x_WO_3_ films (0.10 ≤ x ≤ 0.35). To show the structural changes more clearly, we drew a detailed view of the main diffraction peaks of the sample and the contour of the film. Blue lines represent (002) crystal surfaces and red lines represent (200) crystal surfaces.

In Figure 2, the XRD pattern of WO_3_ embedded with Li^+^ was plotted with details of the peak position change near 24–25°. Contour plots make it more obvious to observe changes in the peak position, peak value, and half-peak width of the plots. By comparison with the JCPDS card No. of 18–0376, the peak at 24.54° corresponds to the (200) crystal plane. As the value of x increases, the peak position is shifted towards a small angle. It decreases from 24.54 to 24.32°. According to Bragg’s Law [31], the insertion of Li^+^ increased the sample cell volume from 423.6 to 434.4, an increase of 2.5%. This results in compressive stress after the sample has been colored [32,33,34].

### 2.2. Ex Situ Raman Spectroscopy

In the monoclinic (Ⅰ) phase, space group P2_1_/*n*($C_{2h}^{5}$), each unit cell contains eight formula units, and the total representation corresponds to 96 normal modes, but these 48 are Raman active: Γ(Raman) = 24A_g_ + 24B_g_. As shown in Table 2, the A_g_ modes have a Raman tensor with non-zero diagonal elements and the xz components (labeled as y on the unique axis). The tensor for the B_g_ modes contains non-zero xy and yz components [35]. Raman spectrum of γ-WO_3_ shows eight vibration modes. Raman peaks at 713.2 and 805.2 cm^−1^ are attributed to O-W-O and W=O stretching vibrations. The peaks at 567.2 cm^−1^ and 625.0 cm^−1^ can be seen by comparing the FTO substrate. In the Raman spectrum of the FTO, the two peaks were characteristic peaks of SnO_2_ [36,37]. The O-W-O bending pattern of the bridging oxide ion peaks appears at 271.0 cm^−1^ and 328.3 cm^−1^ [38]. The peak at 181.1 cm^−1^ and 132.4 cm^−1^ corresponds to the (W_2_O_2_)*_n_* chain [39,40].

**Figure 3:**
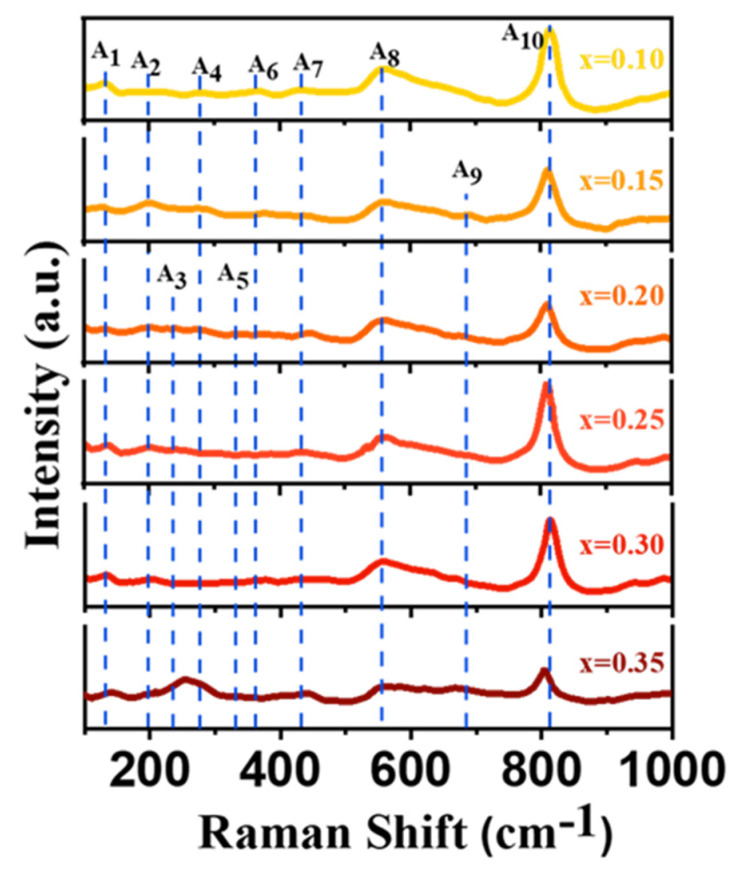
The Raman spectra of the Li_x_WO_3_ (0.10 ≤ x ≤ 0.35).

It causes a strong decrease in the total Raman intensity due to optical absorption after coloring WO_3_ [41]. Additionally, the structural changes associated with the variable lithium content also induce strong changes in the intensity ratio between the various bands. Figure 3 shows the Raman spectrum of Li_x_WO_3_ films with various amounts of lithium insertion and have ten vibration modes. The ten peaks are listed in Table 3. By comparing the Raman spectra of γ-WO_3_ and Li_x_WO_3_, we found three new modes for A_3_, A_6,_ and A_7_ in addition to the seven modes that were affected by lithium insertion in the peak positions. The new modes A_6_ and A_7_ have been identified due to vibrations of the O-W^5+^-O and W^5+^=O bonds, respectively [42,43,44]. When Li^+^ and electrons are inserted into γ-WO_3_ films, the electrons reduce W^6+^ ions to W^5+^ and form O-W^5+^-O and W^5+^=O bonds. These bonds are weaker than the bonds involving W^6+^ ions, so their corresponding Raman peaks appear at lower energies than those for the O-W^6+^-O and bonds [45]. It has also been established that the intensities of A_6_ and A_7_ directly correlate with the coloration of γ-WO_3_ films. According to previous reports [46,47], the A_9_ mode can be classified as O-W^6+^-O stretching vibrations, and the A_10_ mode can be classified as W^6+^=O stretching vibrations [48]. In fact, the 713.2 cm^−1^ mode decreases strongly with respect to the 815.9 cm^−1^ mode upon lithium insertion and disappears for ion concentrations lower than the maximum attainable. This behavior corresponds well to the observed quenching of the same mode in a temperature range below the orthorhombic → tetragonal transition in the case of temperature-driven evolution in pure WO_3_ [47]. Here, we focus on the A_10_ mode. The blue shift in the near 810 cm^−1^ peaks decreases with increasing lithiation seems to be in good agreement with their analysis [46]. This implies that the content of O-W^6+^-O is reduced and transformed to O-W^5+^-O, while the shift in the peak phase in the short wavelength direction implies that the insertion of Li reduces the amplitude of the O-W-O bond and produces a deformation in the structural length. However, the sudden red shift in the vibrational modes in Li_0.10_WO_3_ film and Li_0.35_WO_3_ film may be due to the luminescence contribution caused by the change in optical properties during the phase transition [35]. Combined with the peak position results of XRD, there is an increase in theta angle at (120) (−202) and (202), so the W^6+^=O bonds at this point should correspond to these three crystal planes. After Li^+^ insertion, the attenuation of the vibrational mode of γ-WO_3_ at 713 cm^−1^ is obvious, and then the vibrational mode at 805 cm^−1^ is shifted, suggesting that the W^6+^→W^5+^ reaction occurs preferentially in the interior O-W-O, followed by W=O at the surface [49,50].

### 2.3. Ex Situ Spectroscopic Ellipsometry

**Figure 4:**
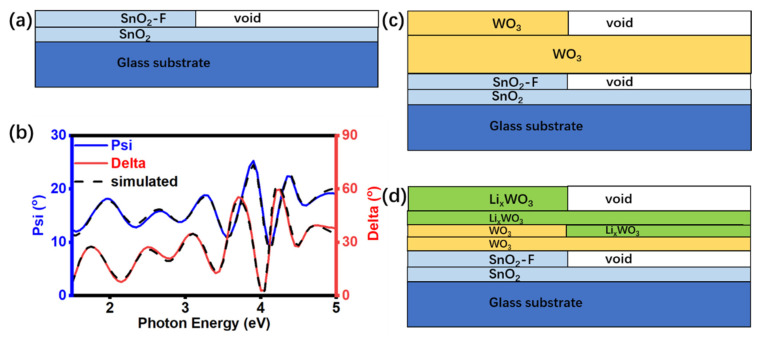
The model of (**a**) the FTO substrate and (**b**) the fit result of it. The model of (**c**) γ-WO_3_ films on the FTO substrate and (**d**) Li_x_WO_3_ films, respectively.

The optical parameters and their evolution in the lithiation processes of Li_x_WO_3_ films were measured and calculated by spectroscopic ellipsometry and DeltaPsi 2 software. As described in the experiment section, the film is deposited on the FTO substrate. To analyze the optical properties of the materials, the optical parameters of the substrate should be determined previously. Commercial FTO substrate is assumed to be a multilayer stack of glass and F-doped SnO_2_ (Figure 4a). A three-phase model of roughness layer/SnO_2_-F bulk layer/glass substrate was built to simulate the optical constants of the FTO substrate. The optical constants for each layer were imported from the Application Library of the software DeltaPsi 2. The corresponding simulated spectra of the FTO substrate agreed well for the entire spectral range with the χ^2^ value of 1.77. The ellipsometric data, Ψ and Δ, measured (solid line) and simulated (dotted line) for FTO, show in Figure 4b.

Spectroscopic ellipsometry results with different Li^+^ insertion dose-dependent were measured to illustrate the optical parameters evolution due to the phase transition pre and post the color change. Figure 4c shows the optical modeling of the γ-WO_3_ film, and Figure 4d shows the optical modeling of the Li_x_WO_3_ film.

### 2.4. Layer-by-Layer Diffusion Mechanism of Li^+^ in γ-WO_3_

**Figure 5:**
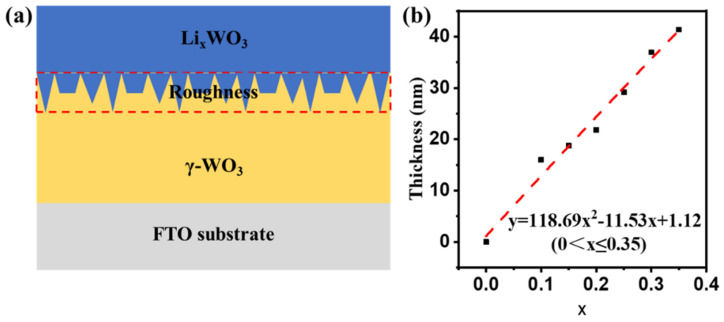
(**a**) Schematic structure of thin film after lithiation. (**b**) Relationship between Li^+^ insertion value x and film thickness.

In Figure 5a, we can consider it as a doped film of γ-WO_3_ and Li_x_WO_3_ for different x values of the films; therefore, calculating the thickness and ratio of the two materials of the γ-WO_3_/Li_x_WO_3_ layer can be used to indicate the Li^+^ insertion dose-dependent of the film. Due to the presence of roughness layer thickness, we added the thickness of the roughness layer in amounts of 50% to each of the two layers. Table 4 shows the fitting thickness results of Li_x_WO_3_ films by the software DeltaPsi 2. We fitted the relationship between x and the ratio of the Li_x_WO_3_ layer to the thickness of the film, as shown in Figure 5b. The relational equation was obtained by polynomial fitting: y = 118.69x^2^ − 11.53x + 1.12 (0 < x ≤ 0.35). When x = 0, there is no Li^+^ insertion, and the thickness of Li_x_WO_3_ is also zero. This case applies only to the coloration of crystalline WO_3_ films in lithium hexafluorophosphate electrolyte. Since the degree of coloration is affected by the crystal structure, specific surface area, cation radius, etc., the establishment of this system requires more research subsequently.

Through the analysis above we can realize that the thickness of the Li_x_WO_3_ layer progressively deepens with the layer-by-layer diffusion of Li^+^ in it. This indicates that the γ-WO_3_ films prepared by the spin-coating method are denser, and the diffusion mechanism of Li^+^ in them is uniform diffusion. Although there is also a rough layer present, there is no particularly obvious ion-preferential diffusion channel. These films are more suitable for applications in the preparation of precision optical components than those prepared by conventional magnetron sputtering [51].

### 2.5. Details of Optical Leaps in the Full Spectrum


(1) $$\frac{d^{2}\left(E^{2}\varepsilon \right)}{dE^{2}}=\left\{\begin{array}{c} {n\left(n-1\right)A_{m}e^{i\phi_{m}}\left(E-E_{cpm}+i\Gamma_{m}\right)}^{n-2}\;n\neq 0\\ {A_{m}e^{j\phi_{m}}\left(E-E_{cpm}+i\Gamma_{m}\right)}^{-2}\;n=0 \end{array}\right.$$

**Figure 6:**
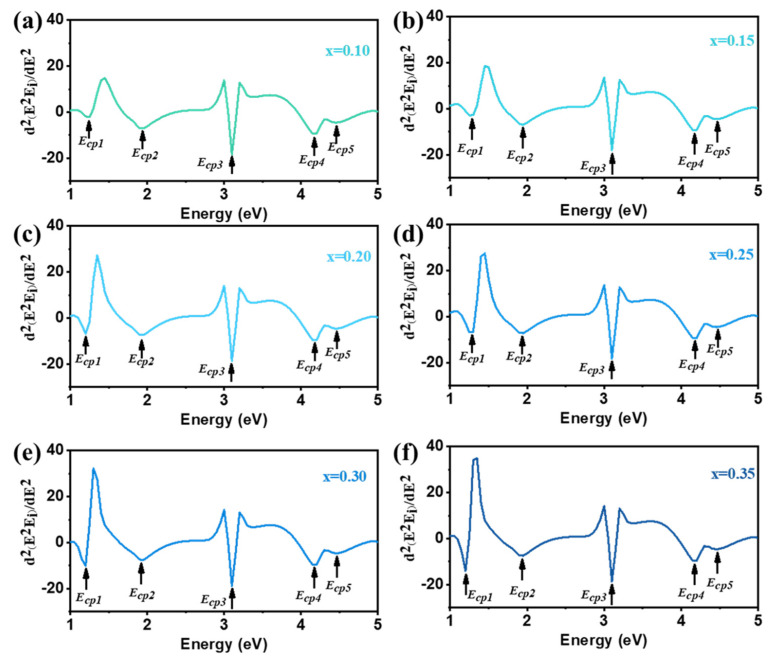
Using the Second derivatives of the pseudo dielectric function numerically calculated spectra of Li_x_WO_3_ (blue) thin films at 300K. The *E*_cp1_, *E*_cp2_, *E*_cp3_, *E*_cp4_, and *E*_cp5_ transition features are indicated by arrows. (**a**–**f**) is the calculated spectra of Li_x_WO_3_ for x = 0.10, x = 0.15, x = 0.20, x = 0.25, x = 0.30, x = 0.35, respectively.

To analyze the optical transitions of Li_x_WO_3_ films, we computed $d^{2}\left(E^{2}\varepsilon \right)/dE^{2}$ in Figure 6. The $d^{2}\left(E^{2}\varepsilon \right)/dE^{2}$ is shown in Equation (1). The positions as well as the shapes of these critical points (CPs) can be totally determined by the CPs analysis method [52]. The transition energies were 2.5 eV, 3.05 eV, 3.65 eV, and 4.55 eV, respectively. We assigned the origins of the four interband transitions according to the results from density functional calculations and previous analysis of the respective materials. The *E*_cp1_ transition arises from a direct gap transition between the highest valence band (HVB) and the lowest conduction band (LCB) at the Γ point in the Brillouin zone (BZ). Another interband transition labeled *E*_cp2_ occurred at the X point in the BZ. The *E*_cp3_ = 3.65 eV transition arises from an indirect gap transition between the HVB at the R point and the LCB at the X point in the BZ. The *E*_cp4_ = 4.55 eV transition arises from a direct gap transition between the HVB and the LCB at the R point in the BZ.

For Li_x_WO_3_ thin film, five distinct transitions labeled *E*_cp1_, *E*_cp2_, *E*_cp3_, *E*_cp4,_ and *E*_cp5_ appear in the spectra (*n* = 1/2). By comparing the data in Table 5, we can know that the CPs of Li_x_WO_3_ films are identical. The transition energies for the Li_x_WO_3_ thin film were 1.2 eV, 1.95 eV, 3.1 eV, 4.2 eV, and 4.45 eV, respectively. The *E*_cp1_ = 1.2 eV transition arises from a direct gap transition between the highest valence band (HVB) and the lowest conduction band (LCB) at the Γ point in the Brillouin zone (BZ). Another interband transition labeled *E*_cp2_ occurred at the X point in the BZ. At the same time, the contribution from the indirect transition of the HVB at the M point and the LCB at the Γ point in the BZ is also included. The *E*_cp3_ = 3.1 eV transition arises from an indirect gap transition between the HVB at the X point and the LCB at the M point in the BZ. The *E*_cp4_ = 4.2 eV transition arises from a direct gap transition between the HVB and the LCB at the M point in the BZ. The *E*_cp5_ = 4.45 eV transition arises from a direct gap transition between the VB and the CB at the R point in the BZ. In all cases, the LCB and HVB are formed by hybridization of W 5*d* and O 2*p* states, but the CB is mainly dominated by W *d* states while the VB has an O *p* character [53]. According to the Burstein–Moss shift effect, when Li is inserted, the sample shows metallicity and the spectral change drastically shifts the Fermi level to CB and slightly reduces the size of the band gap. This effect is typical of a simplex *n*-type semiconductor, where the semiconductor becomes metallic when the doping level is very high. In metallic materials, the Fermi level is in the CB, and this part becomes a partially filled conduction band (PFCB); the band below this one is the highest fully occupied band (B_−1_), and the band above is the lowest unoccupied band (B_1_) [54]. The W 5*d* states dominate both the PFCB and the B_1_, whereas the B_−1_ presents an O 2*p* character number of states. The Li 2*s* states lay beyond the Fermi level higher than the W 5*d* states. The 2*s* level of atomic Li is higher in energy than the 5*d* level of atomic W, and thus, one would expect that the W 5*d* states lay closer to the Fermi level. The position of the Li 2*s* states also helps us elucidate the role played by the Li atom. Since these states lie outside the Fermi energy level, the corresponding valence electrons enter the PFCB (more precisely, the t_2g_ conduction band), thus leaving the Li atom in the ionized state [55]. This suggests that the insertion of Li provides the system with roving electrons, which is consistent with the metallicity reported experimentally [56]. Thus, the metallic transition reoccurs due to the injection of electrons into the system (electron doping) [57]. This is the main reason why the Tauc–Lorentz and Drude models were selected for analytical modeling.

The analysis of the Brillouin zone point locations and details is crucial for the design of optical materials and devices and is important for optimizing the structure of devices such as optical fibers and optical waveguide devices.

### 2.6. Full Spectrum Properties

In Figure 6, the second-order derivative curves of the imaginary part of the dielectric constant are essentially the same, except for the peak intensity of the peak near 1.3 eV, where a significant difference can be observed. As x increases, the intensity of the peak here increases as well. This is because the absorption of the film in the near-infrared band gradually increases as the value of x increases. We have tested the infrared absorption of the sample below and compared its absorption intensity in the infrared band. And it is consistent with its trend.

**Figure 7:**
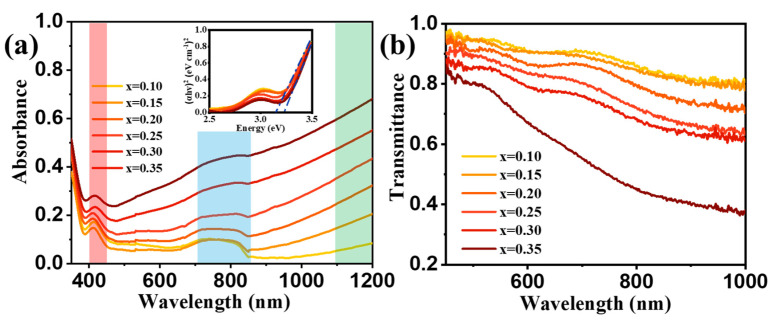
(**a**) The absorption and (**b**) transmission spectra for different values of x. The illustration is the optical bandgaps by simulated. Absorption peaks in the red area is attributed to the absorption of W (VI) (d^0^). Absorption peaks in the blue area is attributed to the absorption of W (V) (d^1^). Absorption peaks in the green area is attributed to the absorption of W (IV) (d^2^).

To observe the change in optical constants during the film color change, we tested the absorption for different values of x, as shown in Figure 7a. It is shown in previous studies that tungsten exists in WO_3_ electrochromic films in tetra-, penta-, and hexavalent forms, and the ratio of each valence state is related to the structure of the material and the color-changing conditions. It is recognized that the hexavalent states of tungsten (d^0^) exhibit charge transfer UV absorption only, while the rest of the valences W^5+^ (d^1^) and W^4+^ (d^2^)show d-d transitions in the visible region [58]. In the absorption spectrum, we can observe two distinct peaks, one near 420 nm and one between 600 and 700 nm. Based on the above findings, the absorption spectra of the films were designated as follows: (a) The UV absorption of the film at 420 nm is attributed to the absorption of W (VI) (d^0^). (b) The broad band centered at 650–800 nm is attributed to W (V) (d^1^). (c) The presence of significant absorption in the near-infrared band is due to the contribution of W (Ⅳ) (d^2^). The peak of 650–800 nm is not easily identifiable in the absorption spectrum, which is caused by the change in the light source in the spectrometer. However, we found a similar peak in the simulated spectrum later. In the transmission spectrum of Figure 7b, the transmittance decreases with increasing x-value, while the color becomes darker. Direct allowed optical transitions were obtained following the expression $\alpha =\frac{A}{hv}{\left(hv-E_{g}\right)}^{\frac{1}{2}}$, where *A* is the photon energy-independent constant, hv is the photon energy, and *E_g_* is the optical bandgap [59]. Optical bandgaps decrease from 3.22 eV to 3.14 eV were estimated of the hybrid Li_x_WO_3_ and γ-WO_3_ films in the illustrations of Figure 7a, respectively, by extrapolating the square of α plots to the energy intercept and are in good agreement with previously reported values [60]. It is influenced by both γ-WO_3_ and Li_x_WO_3_ films together.

Due to the high light modulation ability in the visible light band of Li^+^ insertion in WO_3_ and the strong absorption in the near-infrared band, it has attracted a great deal of attention in the direction of color-changing and heat-modulated glasses in recent years. Meanwhile, the high transmittance in the initial state for color change is also the main reason for its application in the field of smart windows and low power displays [61,62,63].

### 2.7. Optical Constants for Different Li^+^ Insertions

In the electrochromic process of Li^+^ insertion in crystalline WO_3_, the resulting Li_x_WO_3_ films are the same substance and do not change with the value of x. It is only with the different amount of Li^+^ insertion that it participates in the reaction in different proportions, leading to the color change in the films. The optical constants of the resulting Li_x_WO_3_ films are only affected by the ratio of reacted Li_x_WO_3_ to unreacted WO_3_ and by the roughness layer we mentioned above.

**Figure 8:**
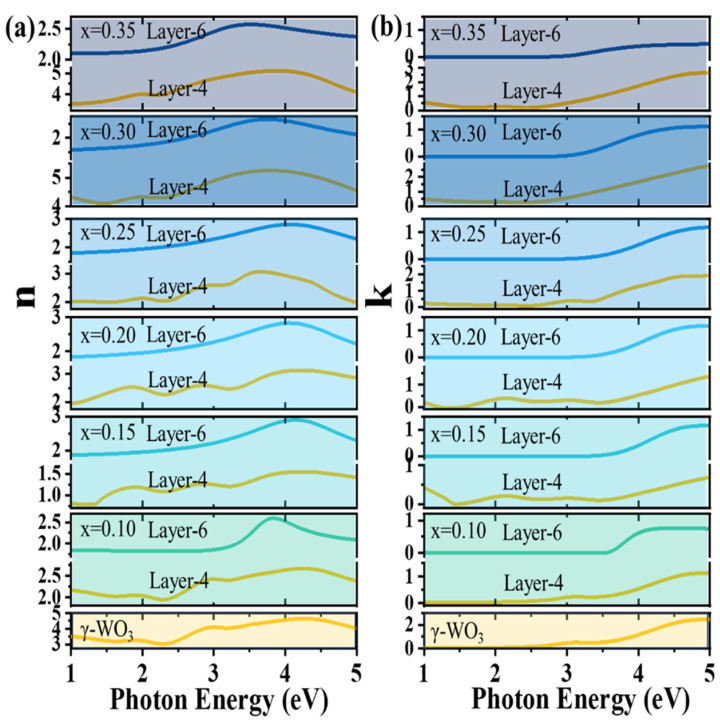
(**a**) The refractive index and (**b**) extinction coefficient of the Li_x_WO_3_ film for different x values. As a comparison, the values of the γ-WO_3_ layer are also given. In fitting Li_x_WO_3_ layers, the optical constants of the γ-WO_3_ layer are fixed.

We also obtained the entire optical and dielectric constants of the film for different values of x. As a result of the fits, the imaginary part of the dielectric function (ε_2_ = 2 nk), which represents optical absorption, is composed of two parts. Figure 8 shows the complex optical constants of the Li_x_WO_3_ film for different x values (the refractive index, *n*, and extinction coefficient, k). The yellow line is the *n* and k for γ-WO_3_ with the value of 0.1 to 0.35 in layer 4. Due to the diffuse reflective influence of the roughness layer, there will be a slight difference from γ-WO_3_, but the overall trend is the same. The refractive index is relatively high in the vicinity of 3–5 eV, i.e., its refractive index is higher in the near UV-blue band at 248–413 nm. There are also smaller shoulder peaks near 1.8 and 2.8 eV, suggesting that it also has a relatively high refractive index for peaks near 450 nm and 690 nm.

We have obtained “pseudo” complex optical constants in this work. As the value of x increases, *n* gradually decreases, and the corresponding photon energy at the non-zero of k decreases. The shift in k represents a shift in the band gap as x increases, decreasing from 3.65 eV to 2.75 eV. However, the roughness layer mainly affects the real part of the complex optical constants rather than the imaginary part. The pseudo-extinction coefficient is reasonably equal to the intrinsic extinction coefficient of almost all spectral features containing inter-band transitions. The extinction coefficient of the film shows a near-monotonic increase with increasing photon energy. The refractive indices of the samples have more pronounced variations around 1.2 eV, 1.8 eV, 3.0 eV, and 3.8 eV, indicating more pronounced modulation of the refractive indices of the light sources at 980 nm, 688 nm, 405 nm, and 325 nm wavelengths. Therefore, it can also be used to prepare optical components such as optical phase modulators, filters, polarizers, etc. It also has the potential to be applied in the scenarios of chip fabrication [64,65], medical imaging [66], and medical therapy [67]. The ex situ spectroscopic ellipsometry provides us with the optical constants of the full spectrum of the samples in various states, which provides more accurate information for the study of the properties and applications of the materials.

**Figure 9:**
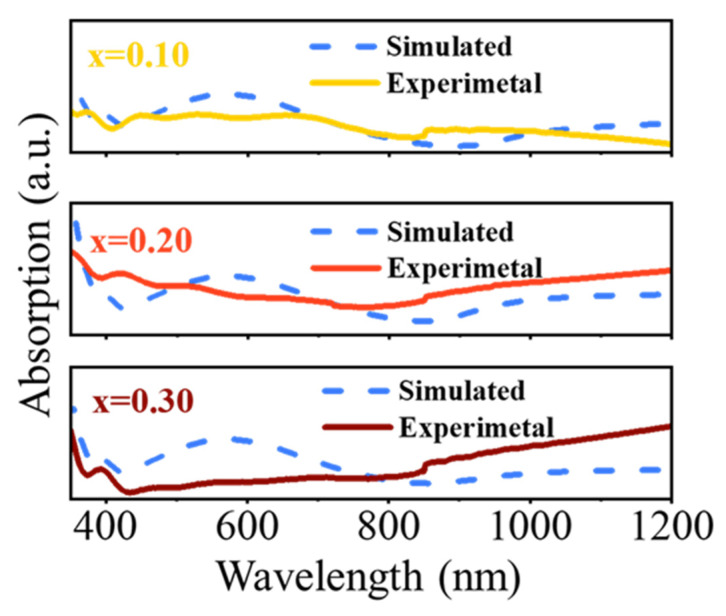
Simulated and experimental absorption coefficient spectra of thin films on the FTO substrate in the visible region with x = 0.10, 0.20, and 0.30.

We confirmed the validity of the spectroscopic ellipsometry analysis by comparing the experimental and simulated absorption spectra [68]. Figure 9 shows the absorption coefficient α spectra and the transmittance intensity of the thin film samples obtained from the experiment, while the dashed line shows the spectrum calculated by SE modeling. The simulated spectra were calculated by fitting the values of the extinction coefficients obtained. The absorption coefficients of the thin films are obtained from $\alpha =\frac{4\pi k}{\lambda }$, where α is the absorption coefficient, k is the extinction coefficient, and λ is the wavelength of light in a vacuum [69,70,71]. Spectra obtained via the method is matched well. The peak values are slightly deviated, which may be a result of the roughness between γ-WO_3_ and Li_x_WO_3_.

## 3. Discussion

In summary, we developed a systematic protocol of ex situ methods to clarify the evolutions of subtle structure changes, Raman vibration modes, and optical parameters of WO_3_ thin films in electrochromic processes as stimulated by dosage-dependent Li^+^ insertion. The crystal structure of the samples gradually transforms the γ → β → α-phase during the coloration process. We proved the layer-by-layer Li^+^ insertion mechanism by analyzing the film thickness of each layer. The relationship between the Li^+^ insertion dosage and the thickness of Li_x_WO_3_ films was obtained: y = 118.69x^2^ − 11.53x + 1.12 (0 < x ≤ 0.35). The detail of all optical leap points in the Brillouin zone corresponding to the range of 1–5 eV is also found. The change in extinction coefficient (k) with Li^+^ insertion shows that its band gap decreases from 3.22 eV to 3.14 eV. The refractive index of each film layer was also used to calculate the relationship between the variation in optical phase angle with wavelength at different Li^+^ insertion dosages. This work provides new ideas for controllable preparation and smart applications of smart optical films.

## 4. Methods

### 4.1. Chemicals and Materials

The tungsten powder (W, 99.80%) and hydrogen peroxide (H_2_O_2_, 30%) were purchased from Sinopharm Chemical Reagent Co., Ltd. (Shanghai, China). Lithium hexafluorophosphate (LiPF_6_) electrolyte purchased from Dongguan Koluder Innovation Technology Co., Ltd. (Dongguan, China). Specification of the electrolyte: 1.0 mol LiPF_6_ in ethylene carbonate (EC): dimethyl carbonate (DMC): ethyl methyl carbonate (EMC) = 1:1:1. Analytical purity acetone, isopropyl alcohol, and ethanol were purchased from Beijing Chemical Industry (Beijing, China). Fluorine-doped tin oxide (FTO) substrates (size 2 × 2 cm^2^) with a surface square resistance of 15 Ω/□ were purchased from Advanced Election Technology Co., Ltd. (Liaoning, China).

### 4.2. Films Preparation

The WO_3_ film was fabricated via the spin-coating method: (1) substrate pretreatment. FTO substrates were cleaned in the solvents of acetone, isopropyl alcohol, ethanol, and deionized water for 15 min subsequently. The adhered solvent molecules were dried by a high-pressure gas gun, and then, the substrates were enclosed in argon gas-filled ultraviolet ozone cleaner for 15 min to remove the organic residues attached to the surface of the substrate and to improve the hydrophilicity of the surface with more of dangling polar chemical bonds. This protocol is very important for the spreading of the precursor solutions to the homogeneous films. (2) Spin coating of WO_3_ films. The precursor solutions should be prepared in advance. Firstly, 2 g W (tungsten powder, 99.8%) was added to 10 mL H_2_O_2_ (hydrogen peroxide, 30%) several times, and their reaction was accelerated at 70 °C; the solution was at a speed of 300 rpm mixed with 2 h at 70 °C and filter with water-based filter head to prepare a saturated solution of metatuntungstic acid. Next, we used a platinum filament to decompose the uncreated hydrogen peroxide and added absolute ethyl alcohol to it. It was allowed to evaporate for 0.5 h at 80 °C and then we obtained the orange transparent colloidal sol. The substrates were mounted on the spinner by vacuum, and then 100 μL of the precursor solutions were dripped onto the substrates. (3) Thermal treatment. The as-prepared WO_3_ film precursor solutions were spin-coated on an FTO substrate at a speed of 3000 rpm for 30 s, followed by annealing at 500 °C for 2 h. Then, the films were cooled to room temperature naturally.

Based on the above experiments, we prepared Li_x_WO_3_ films with different lithium-ion insertion amounts. We used a three-electrode method to color the films. The counter electrode using Pt is the most stable [72]; the Pt sheet is parallel to the WO_3_ film, the working electrode is WO_3_ film, and the reference electrode is Ag/AgCl. The amount of lithium embedded is controlled by the voltage and time applied to the film. We applied “1.5 V, 1 s”, “2 V, 1 s”, “2.5 V, 1 s”, “3 V, 1 s”, “3.5 V, 1 s”, “4 V, 1 s” and “4 V, 10 s” to the obtained WO_3_ films in Multi-potential step method of the electrochemical workstation, respectively. After removal, the sample surface was rinsed with alcohol and blown dry. With the data from the electrochemical workstation, we calculated the insertion levels of Li ions and obtained the films with different insertion levels. the “Li_0.10_WO_3_”, “Li_0.15_WO_3_”, “Li_0.20_WO_3_”, “Li_0.25_WO_3_”, “Li_0.30_WO_3_”and ““Li_0.35_WO_3_” corresponding to ca. 13, ca. 28, ca. 47, ca. 64, ca. 79, and ca. 93 mC/cm^2^ of charge, respectively [73].

### 4.3. Characterization

The characterization details were described as follows: (1) Structure. The phases of the films were determined by x-ray diffraction (XRD) technique with all the data collected from a Rigaku D/max−2500 (Tokyo, Japan) diffractometer with CuKα radiation (λ = 0.15406 nm) operating at a beam voltage of 40 kV and current of 200 mA, and a diffraction angle step of 0.02° in the angle range of 5–50°. (2) Raman spectroscopy. The Raman spectra were recorded using a HORIBA LabRAM HR Confocal Raman microscopy (Kyoto, Japan). The spectra were collected in the range of 50–1000 cm^−1^ at a spectral resolution of 1 cm^−1^ laser excitation with Spectra Physics 473 M ion laser (Kyoto, Japan). (3) Electrochromic performance. The visible transmittance/absorption spectra were measured using a StellarNet Black-Comet-SR Series fiber spectrometer (Tampa, FL, USA). The standardization of the lithiation process of thin films is achieved with the CHI 760E electrochemical workstation from CH Instruments Inc. (Shanghai, China). This is achieved by controlling the excitation voltage and time. The measurements were preceded by five activation cycles in the same electrolyte. The test range in LiPF_6_ electrolyte is −0.3 V to 0.8 V; the scan rate is 100 mV/s.

### 4.4. Optical Properties

Spectroscopic ellipsometry is a simple, non-invasive, surface-sensitive technique based on measuring the relative phase change in reflected and polarized light from a sample to characterize the thin-film optical constants and other properties (e.g., thickness, interface conditions, and roughness) [31,32,33]. To measure the optical constants and dispersion of WO_3_ film during Li^+^ ion migration, the ellipsometry data were recorded as a function of the photon energy in the range of 1.5–5 eV by a HORIBA Jobin yvon UVISEL (Kyoto, Japan) spectroscopic ellipsometry system equipped at an incident angle of 70° in air, and the I_s_ = sin2ΨsinΔ and I_c_ = sin2ΨcosΔ. The ellipsometry data were acquired with different degrees of Li^+^ ion insertion. The optical parameters were obtained by simulating the measured wavelength/energy dependent Delta/Psi curves by the modeling in the DeltaPsi 2 software. The inversion of the ellipsometric data was performed using a five-phase representative model of the samples that added an EC active layer and roughness layer on the model of FTO. In this model, the thickness of the roughness layer, the thickness of the active layer, and the dielectric function of the active layer were unknown, while the roughness layer was modeled by a mixture of 50% air and 50% active material according to the Bruggeman effective-medium approximation (EMA) [74,75,76,77]. The unknown parameters were determined by minimizing the mean squared difference between the generated Is and I_c_ data and the experimental data. All solutions were found to be Kramers−Kronig consistent [78].

## Figures and Tables

**Table 1:** The XRD pattern details the specific peak of Li_x_WO_3_.

x (Li)	0	0.10	0.15	0.20	0.25	0.30	0.35
1	23.16	23.10	23.06	23.14	23.12	23.0	23.0
2	23.66	23.5	23.54	23.56	23.5	23.48	23.5
3	24.34	24.3	24.22	24.14	24.18	24.14	24.14
4	26.52	26.56	26.52	26.54	26.52	26.5	26.58
5		——	——	——	——	——	28.26
6	28.76	28.82	28.66	28.8	28.72	28.66	28.72
7	33.48	33.66	33.58	33.62	33.6	33.56	33.62
8	——	——	33.94	33.96	34.02	33.95	33.86
9	34.2	34.18	34.22	34.24	34.24	34.26	34.28
10	37.8	37.78	37.76	37.0	37.8	37.78	37.78

**Table 2:** Raman peak position and associated vibrational modes of γ-WO_3_.

Raman Peak Position (cm^−1^)	Peak Assignments
132.4, 181.1	Lattice vibration mode
271.0, 328.3	δ (O-W-O), bending vibration modes
567.2, 625.0	attributed to FTO
713.2, 805.2	ν (O-W-O/W=O), stretching vibration modes

**Table 3:** Raman peak position for the Li_x_WO_3_ films.

x (Li)	0.10	0.15	0.20	0.25	0.30	0.35
A_1_ (cm^−1^)	130.1	130.1	132.4	137.1	137.1	141.7
A_2_ (cm^−1^)	197.3	199.6	201.9	201.9	204.3	204.3
A_3_ (cm^−1^)	——	——	241.2	241.2	248.1	254.9
A_4_ (cm^−1^)	277.9	282.5	275.6	307.7	323.7	325.9
A_5_ (cm^−1^)	——	——	335.1	348.8	364.7	351.0
A_6_ (cm^−1^)	369.6	376.1	373.7	387.4	378.3	398.7
A_7_ (cm^−1^)	430.4	439.4	446.2	430.4	437.2	439.4
A_8_ (cm^−1^)	567.2	567.2	567.2	567.2	567.2	567.2
A_9_ (cm^−1^)	——	691.3	678.05	673.6	667.0	667.0
A_10_ (cm^−1^)	813.7	811.5	809.4	809.4	809.4	802.8

The sign “—” denotes that no peak could be read for the possible vibration mode in the specific Raman spectrum for the materials.

**Table 4:** Fitting thickness results of Li_x_WO_3_ films by DeltaPsi 2.

x (Li)	0	0.10	0.15	0.20	0.25	0.30	0.35
Li_x_WO_3_ (nm)	——	16.01	18.74	21.82	29.14	36.94	41.35
γ-WO_3_ (nm)	380	364	361.42	358.22	351.83	343.06	338.66

We added the thickness of the roughness layer in amounts of 50% each to each of the two layers.

**Table 5:** Trip thresholds for γ-WO_3_ (yellow) and Li_x_WO_3_ (blue) films calculated from the second derivative of the pseudo-dielectric constant.

x (Li)	0	0.10	0.15	0.20	0.25	0.30	0.35
*E*_cp1_	——	1.2	1.2	1.2	1.2	1.2	1.2
*E*_cp2_	2.5	1.95	1.95	1.95	1.95	1.95	1.95
*E*_cp3_	3.05	3.1	3.1	3.1	3.1	3.1	3.1
*E*_cp4_	3.65	4.2	4.2	4.2	4.2	4.2	4.2
*E*_cp5_	4.55	4.45	4.45	4.45	4.45	4.45	4.45

## Data Availability

The data that support the findings of this study are available from the authors upon reasonable request.
